# Household disaster preparedness and information sources: Rapid cluster survey after a storm in New South Wales, Australia

**DOI:** 10.1186/1471-2458-8-195

**Published:** 2008-06-04

**Authors:** Michelle Cretikos, Keith Eastwood, Craig Dalton, Tony Merritt, Frank Tuyl, Linda Winn, David Durrheim

**Affiliations:** 1 NSW Public Health Officer Training Program, Centre for Epidemiology and Research, NSW Department of Health, New South Wales, Australia; 2 Population Health Unit, Hunter New England Area Health Service, Newcastle, New South Wales, Australia; 3 School of Medical Practice and Population Health, University of Newcastle, Newcastle, New South Wales, Australia; 4 Hunter Medical Research Institute, Newcastle, New South Wales, Australia; 5 Disaster Response and Coordination Unit, Hunter New England Area Health Service, Newcastle, New South Wales, Australia

## Abstract

**Background:**

A storm-related disaster in New South Wales, Australia in June 2007 caused infrastructure damage, interrupted essential services, and presented major public health risks. We investigated household disaster preparedness and information sources used before and during the disaster.

**Methods:**

Rapid cluster survey of 320 randomly selected households in Newcastle and Lake Macquarie, New South Wales, Australia.

**Results:**

227 households (71%) responded to the survey. By the day before the storm, 48% (95%CI 40–57%) of households were aware of a storm warning, principally through television (67%; 58–75%) and radio (57%; 49–66%) announcements. Storm preparations were made by 42% (28–56%) of these households.

Storm information sources included: radio (78%; 68–88%); family, friends, colleagues and neighbours (50%; 40–60%); and television (41%; 30–52%). Radio was considered more useful than television (62%; 51–73% vs. 29%; 18–40%), even in households where electricity supply was uninterrupted (52%; 31–73% vs. 41%; 20–63%).

Only 23% (16–30%) of households were aware that the local government-operated radio network has a designated communication role during disasters. A battery-operated household radio and appropriate batteries were available in 42% (34–50%) of households, while only 23% (16–29%) had all of: a torch, battery-operated radio, appropriate batteries, mobile phone, emergency contact list and first aid equipment.

**Conclusion:**

Broadcast media are important information sources immediately before and during disasters. Health services should promote awareness of broadcast networks' disaster role, especially the role of radio, and encourage general household disaster preparedness. A rapid cluster survey conducted shortly after a natural disaster provided practical, robust information for disaster planning.

## Background

**Figure 1:**
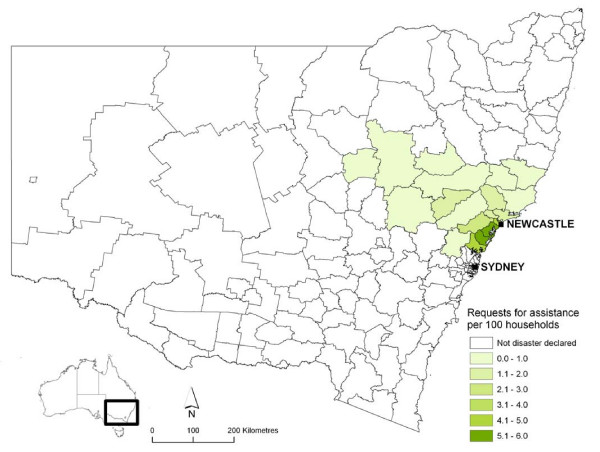
Requests for assistance from the New South Wales State Emergency Service per 100 households in local government areas declared natural disaster areas as a result of the storm in June 2007.

A severe storm that began on Thursday, 7 June 2007 brought heavy rains and gale force winds to the Newcastle, Central Coast and Sydney regions of New South Wales, Australia (Figure 1). At least ten people died as a direct result of the storms, including a family of five who died when a section of highway collapsed and a couple who died when their car was swept off a bridge.

Rainfall of up to 275 mm in 24 hours, and wind gusts exceeding 130 km/hour. [1] caused widespread flooding and damage to houses, businesses, schools, hospitals, nursing homes and community health centres. Local infrastructure was severely affected, resulting in power, water and gas supply interruptions; sewerage system failures; and rail line damage. Many roads were impassable due to floodwater, fallen trees and power lines, and abandoned cars.

The State Emergency Service responded to almost 20,000 storm-related requests for assistance [2], while widespread flooding resulted in evacuation of over 6000 residents. The failure of sewage and water utility pumps resulted in contamination of flood water, as well as difficulty in ensuring adequate quality and quantity of drinking water. A natural disaster was declared for a total of 19 local government areas with a population of over 1 million people (Figure 1). [2] The total storm damage bill is expected to reach A$1.5 billion [3].

It is well understood that the effectiveness of public communication strategies and level of community disaster preparedness can determine the success of a disaster response. [4-8] While there are recommendations for household disaster preparedness in Australia, very little is known about the actual level of household disaster preparedness, or household information needs and information sources used during a disaster, although radio networks have been identified as important information sources during bushfires and other emergency situations [9,10].

Anecdotal reports suggest that access to information during the June storm was hampered by power failures, a lack of battery operated radios, and lack of community awareness of radio networks' role in providing emergency information. In the context of this natural disaster, the aim of the survey was to investigate household disaster preparedness, emergency radio network awareness, household information needs and information sources accessed by households during the disaster.

## Methods

### Study design

**Figure 2:**
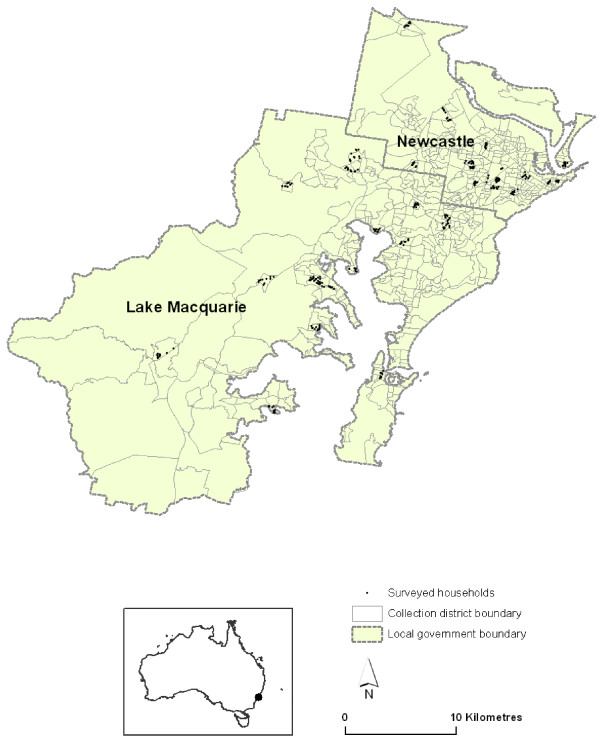
Location of households randomly selected from the Newcastle and Lake Macquarie local government areas of New South Wales.

A two-stage cluster sample design was used. The primary sampling unit was the census collection district, and the unit of analysis was the household. The list of collection districts and household addresses was obtained from the 2001 Australian Census. We estimated that for a cluster size of 10 households, we would need 30 clusters to achieve acceptable precision. We randomly selected 32 collection districts from two of the worst affected local government areas within our Area Health Service: Newcastle and Lake Macquarie. We randomly selected 15 household addresses from within each collection district to ensure that 10 valid addresses were available to achieve a sample of 320 households (Figure 2).

### Survey distribution and collection

The survey instrument was piloted on health services staff before use. We visited randomly selected households during the first and second rounds of survey distribution. Households were excluded if they were found to be commercial properties, vacant lots, uninhabited, non-existent or if no-one from the household had sufficient English to complete the survey. The next randomly selected household address was visited until 10 surveys had been successfully distributed in each collection district.

We asked households to select the householder aged 18 years or more who was most able to complete the survey on behalf of the household. In order to maximize the response rate, up to two home visits were made, and householders were also given the opportunity to complete the survey themselves and return it by post. Five survey teams delivered all surveys within two weeks of the storm and completed a face-to-face interview where possible.

**Figure 3:**
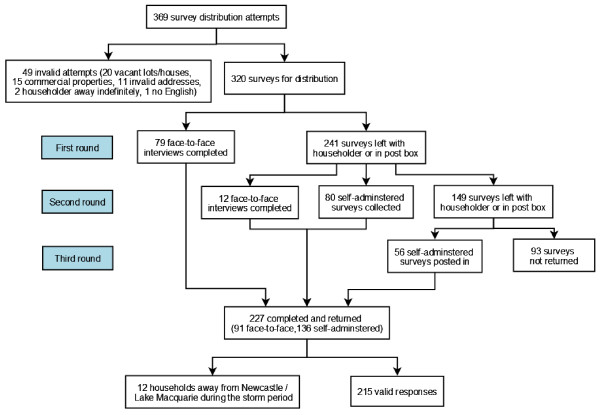
Distribution of surveys.

If a householder was not at home at the time of the first visit, the survey was left in the letterbox with a reply-paid envelope provided. A minimum of two days after the first visit the survey teams revisited homes to collect completed surveys and to administer further face-to-face interviews where possible. Households that still had not completed a survey after the second visit were given a reminder to complete and return their survey using the reply-paid envelope provided. A summary of the distribution method and responses is provided in Figure 3.

### Mapping

We used ArcMap version 9.2 (ArcGIS by ESRI Inc., Redlands, California, USA) to map the severity of the storm in the disaster affected areas. Storm severity was assessed using rates of requests for assistance to the NSW State Emergency Service per 100 households, which were calculated using NSW State Emergency Service request for assistance data and local government area data from the 2006 Australian Census.

### Statistical analysis

To minimise error, data were double-entered into a purpose-designed Microsoft Access database. Household representativeness was assessed by comparison to the 2006 Australian Census. [11] Data were analysed using Stata statistical software (Stata Version 10.0, Stata Corp, College Station, Texas, USA). Households that reported they were away from the Newcastle or Lake Macquarie region during the storm period were excluded. Households without the relevant service connection were excluded from service interruption estimates. All estimates included missing responses in the denominator. Point estimates were adjusted using sampling weights, while confidence intervals were adjusted for the clustered design effect using Taylor-linearised variance estimation. Results are reported with 95% confidence intervals.

This study was approved by the Area Health Service Chief Executive and conducted as part of the disaster response. Ethics committee approval was not required.

## Results

### Survey distribution and collection

A total of 369 survey distribution attempts were required to successfully distribute 320 surveys (Figure 3). Reasons for distribution failure included: vacant lot or vacant house (20 attempts, 5.4%), commercial property (15 attempts, 4.0%), address did not exist (11 attempts, 3.0%), householders were away indefinitely (2 attempts, 0.5%) or could not speak English (1 attempt, 0.3%). Overall 227 of the 320 (70.9%) surveys were completed and returned, of which 91 (40.1%) were face-to-face interviews and 136 (59.9%) were self-administered.

### Respondent demographics

**Figure 4:**
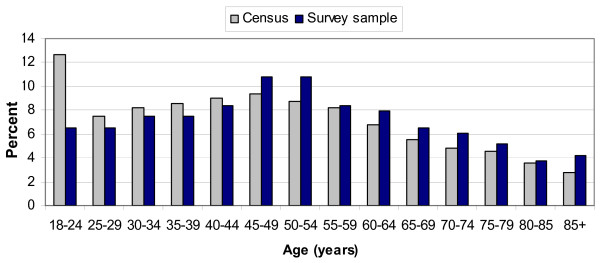
Age distribution of survey respondents present during the storm period.

Of respondents, 94.7% (215/227) were in the Newcastle or Lake Macquarie region during the storm period of 8 to 9 June 2007. Respondents ranged from 19–90 years old, with a mean of 51 years. Most age groups were well represented (Figure 4).

Female respondents were over-represented, making up 65% of respondents compared to 52% of the study population. [11] The sample had a mean of 2.7 people per household, compared to a mean of 2.6 for the study population [11].

### Impact of the storm

Flood water entered 18.6% of houses (95% CI 12.0–25.2%) and 2.9% (0.7–5.1%) of houses were temporarily or permanently vacated. Car damage was reported by 9.0% (4.1–13.8%) of households, while 1.6% (0.0–3.8%) of households reported a storm related injury of some kind. These injuries were generally minor.

The storm caused a number of major service interruptions. 73.9% (59.4–88.3%) of households experienced electricity service interruption. 20.6% (9.7–31.5%) reported electricity interruption for 48 hours or more. 43.4% (32.0–54.8%) of households with a landline telephone connection reported that this service was interrupted, and 41.2% (31.2–51.1%) of households with a mobile phone experienced service interruption.

A number of households (14.5%; 8.7–20.2%) attempted to access cash during the storm period. Cash sources included automatic teller machines (ATMs, 44.2%), electronic funds transfer (38.3%) and banks (3.2%). 45.8% (26.4–65.2%) of those who tried to access cash experienced difficulties. These were principally due to ATMs not functioning, shops being closed, and difficulty accessing shops or ATMs because of storm damage.

As a result of the storm, 30.2% (19.9–40.5%) of households received assistance from family, friends or neighbours. In those households that received this assistance, 42.5% received one or more meals, 25% used a fridge, 24.6% stayed overnight, 18.7% loaned equipment or supplies, 13.8% were provided with hot water or hot showers, 12.5% used a washing machine and 11.8% received assistance moving household goods.

State Emergency Service assistance was requested by 4.4% (1.5–7.2%) of households. Some households experienced trouble contacting this service or received a delayed response. Assistance was most commonly requested because of fallen trees and storm damage to houses.

### Household storm preparedness

**Figure 5:**
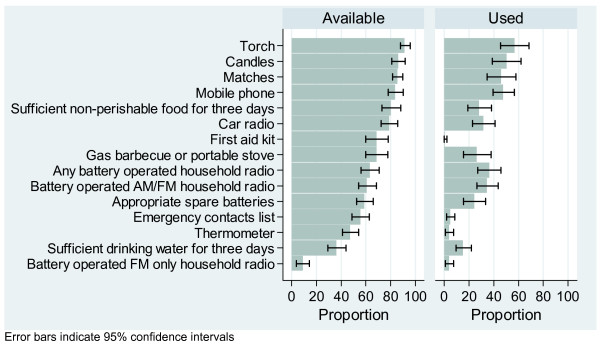
Household disaster preparedness: proportion of households with equipment available before the storm or used during the storm.

Household disaster preparedness was variable. Basic supplies including a mobile phone, a torch, candles, matches and a three day supply of non-perishable food were available in over 80% of households, but other important equipment including household battery-operated radios, appropriate spare batteries, emergency contact lists, first aid kits and thermometers were less commonly available. Less than half of households had sufficient drinking water for three days (Figure 5).

As expected, certain equipment was used significantly more often in households that experienced electricity interruption when compared with those households that did not. This equipment included battery operated radios (44.2%; 34.5–54.0% vs. 15.1%; 6.7–23.5%), torches (72.9%; 64.8–80.9% vs. 11.8%; 3.6–20.0%), spare batteries (32.6%; 23.0–42.1% vs. 1.6%; 0.0–4.8%), candles (65.8%; 56.1–75.4% vs. 6.9%; 0.0–14.0%), matches (61.7%; 52.4–71.1% vs. 2.6%; 0.0–6.5%) and a portable stove (34.2%; 21.0–47.5% vs. 5.5%; 0.0–12.0%).

Only 42.0% (33.9–50.0%) of households had both a battery-operated household radio and appropriate batteries available. Only 22.8% (16.2–29.4%) of households had all of: a torch, battery operated radio, appropriate batteries, mobile phone, emergency contact list and first aid kit. This equipment forms only a part of the recommended household emergency survival checklist [12].

### Storm warning awareness and information sources

**Figure 6:**
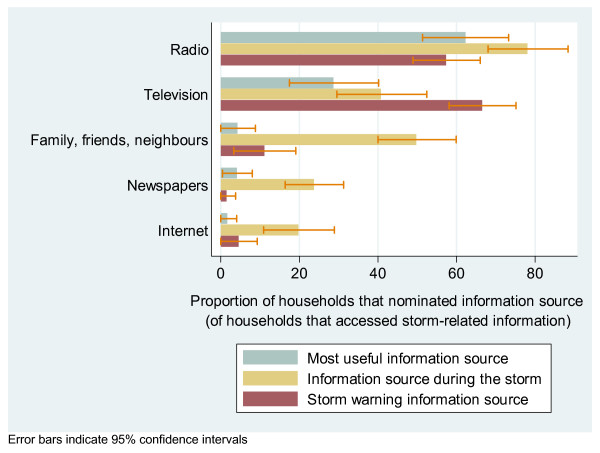
Household storm information sources.

On the day before the storm, 48.1% (39.8–56.5%) of households were aware of a storm warning through television (66.6%; 58.1–75.1%), radio (57.4%; 48.9–66.0%), or family, friends and work colleagues (11.2%; 3.3–19.1%, Figure 6).

Preparations for the storm were made by 41.7% (27.6–55.9%) of households that received a storm warning. Preparations included clearing the yard and drains, securing windows and loose objects, making sure that emergency equipment (e.g., candles and torches) was available and cancelling travel.

Information about the storm or emergency services was accessed by 50.2% (41.6–58.8%) of households during the storm period. The three most common information sources were radio (78.1%; 68.0–88.3%), family, friends, neighbours and work colleagues (49.9; 40.0–59.9), and television (40.9%; 29.5–52.4, Figure 6).

Most householders reported that the radio was the most useful information source. Radio was significantly more useful than the next most useful source, the television. Even in households where electricity supply was not interrupted, radio was still considered more useful than television (52.0%; 30.6–73.4% vs. 41.3%; 19.8–62.9%, Figure 6).

The information most commonly sought by households during the storm period included details on storm damage and weather reports (51.6%; 41.2–61.9%), road closures (41.7%; 32.0–51.5%), and timelines for the restoration of electricity and other essential services (22.5%; 13.6–31.4%). Householders were generally able to find the information they were seeking. Those that tried to access information during the storm reported that information on electricity and other service restoration (13.5%; 6.9–20.0) and road closures (8.4%; 2.3–14.5%) was most difficult to obtain.

An estimated 45.4% (36.8–54.1%) of households listened to the local government-operated radio station, while only 23.1% (15.7–30.4) of householders were aware that this radio station has a designated communications role during emergencies and disasters.

## Discussion

Approximately half of surveyed households were aware of a storm warning by the day before the storm, with both television and radio being important sources of the warning. Almost half of these households took sensible measures to protect themselves and their property. During the storm period, radio replaced television as the most commonly accessed source of information, and was considered the most useful source of information overall, even in households that did not experience electricity interruption. Awareness of the role of the local emergency radio network during disasters was low.

Of concern, less than half of households had the basic equipment necessary – a battery operated radio and appropriate batteries – to receive emergency service messages and warnings during a disaster involving electricity interruption. Even fewer had other recommended household emergency equipment available at home [12].

Our study had a number of strengths, including the use of a randomly selected sample of households surveyed within two weeks of the storm, so that recall of storm events, information needs and equipment used was more likely to be accurate. We achieved a good response rate, and the surveyed households were representative. Although the youngest (18–24 year) age group was under-represented, this was expected, and we believe that this was the result of requesting an adult representative to respond on behalf of the household, with older adults more likely to be selected. This should not have had a substantial impact on our survey estimates, as household experiences and preparedness were the main areas of interest, rather than individual experiences. Similarly, although the survey respondents were more commonly female this should not have had a substantial impact on the responses provided on behalf of the household.

This study covered only two of the affected local government areas and the results may therefore not be readily generalisable to all affected areas, or to Australia as a whole. In addition, only one kind of natural disaster was examined, and it is possible that the results may not be generalisable to a bioterrorist or infectious disease emergency. This study also did not explore all aspects of household disaster preparedness. Future surveys of this type could explore issues relating to vulnerable sub-groups such as young children and those with chronic illness, and could further explore general household preparedness including: household supplies of prescription medication, appropriateness of household emergency plans, and knowledge of techniques for disinfection of water

The findings from this study are already proving useful for planning for future disasters, both natural and manmade, and have important practical implications for public health emergency policy and practice. Firstly, Australian emergency plans nominate emergency warnings through radio networks as one of the main strategies for emergency public communication, particularly for rapidly evolving emergencies or disasters involving electricity interruptions. [10,13,14] This approach assumes that households have certain basic equipment such as battery-operated radios available, and that households are aware of the disaster role of radio networks. Our survey indicates that neither of these assumptions are valid for our community, although our findings confirm the importance of radio as a source of information during disasters.

Secondly, although 23% of householders were aware that the local government radio station had a designated role during emergencies, no formal agreement for such a role exists in New South Wales. A national bushfire enquiry in 2003 recommended that all Australian states develop formal arrangements with the national government-operated emergency broadcaster, but this recommendation has only been implemented by three Australian states to date. [10] The results of this survey should help to inform policy development around this issue.

Finally, we believe that rapid cluster surveys could be used more often in emergency or disaster settings, as they provide an opportunity to capture real-time, accurate and representative information about the community impact of a disaster, and the effectiveness of the disaster response.

## Conclusion

A widespread natural disaster which developed rapidly in New South Wales, Australia in June 2007 resulted in substantial infrastructure damage and interruptions to essential services, and posed a serious public health risk. A rapidly conducted household survey identified that emergency radio networks form an important emergency communication tool during disasters, especially when electricity services are interrupted. The study also identified a need to improve the effectiveness of disaster warnings, and to ensure that households have the necessary equipment to allow them to receive emergency messages during a disaster. Health services should consider working with emergency service and broadcast media organisations to promote community disaster preparedness in general and awareness of local emergency radio networks in particular.

## Competing interests

The authors declare that they have no competing interests.

## Authors' contributions

MC designed the study, performed the statistical analysis and drafted the manuscript. KE, TM and LW participated in design and coordination of the study and critically reviewed the manuscript. CD and DD conceived of the study, participated in its design and coordination and critically reviewed the manuscript. FT assisted with the statistical analysis and critically reviewed the manuscript. All authors read and approved the final manuscript.

## Pre-publication history

The pre-publication history for this paper can be accessed here:



## References

[B1] Bureau of Meteorology Newcastle New South Wales June daily weather observations. http://www.bom.gov.au/climate/dwo/200706/html/IDCJDW2097.200706.shtml.

[B2] Emergency Management Australia Disasters Database NSW east coast storm and flood event. http://www.ema.gov.au/ema/emadisasters.nsf/9d804be3fb07ff5cca256d1100189e22/99221b6265ebad62ca2573070025a88c?OpenDocument.

[B3] John D Winter storm bill expected to reach $1.5b. http://www.smh.com.au/news/national/winter-storm-bill-expected-to-reach-15b/2007/08/24/1187462523612.html.

[B4] World Health Organization Effective communication during public health emergencies: A WHO handbook. http://www.who.int/csr/resources/publications/WHO%20MEDIA%20HANDBOOK.pdf.

[B5] Federal Emergency Management Agency National incident management system: Draft August 2007. http://www.fema.gov/library/viewRecord.do?id=2961.

[B6] Australasian Fire Authority Council The Australasian Inter-service Incident Management System: A management system for any emergency. http://www.forestrytas.com.au/forestrytas/fire_management_documents/operational_manuals/other_agency_manuals/incident_managment_system_manual.pdf.

[B7] HM Government Emergency preparedness. Emergency preparedness.

[B8] International Strategy for Disaster Reduction Building disaster resilient communities: Good practices and lessons learned. http://www.unisdr.org/eng/about_isdr/isdr-publications/06-ngos-good-practices/ngos-good-practices.pdf.

[B9] Walker R, Robinson P, Tebbutt J, Lin V, Bissett P, Burns R, Schauble J Emergency management risk communication project: Final report to the Department of Human Services. http://www.health.vic.gov.au/environment/downloads/risk_communication.pdf.

[B10] Ellis S, Kanowski P, Whelan R National inquiry on bushfire mitigation and management. http://www.coagbushfireenquiry.gov.au/findings.htm.

[B11] Australian Bureau of Statistics 2006 Census data online. http://www.censusdata.abs.gov.au/websitedbs/d3310114.nsf/home/Census%20data.

[B12] Emergency Management Australia A checklist for your emergency survival kit. http://www.ema.gov.au/agd/ema/rwpattach.nsf/VAP/(A80860EC13A61F5BA8C1121176F6CC3C)~PFTU_checklist2007.pdf.

[B13] Emergency Management Australia Guide 5: Flood warning. Second edition. Australian Emergency Manuals Series Part III: Emergency Management Practice.

[B14] Australian Government Department of Health and Ageing Australian health management plan for pandemic influenza. http://www.health.gov.au/internet/main/publishing.nsf/Content/ohp-pandemic-ahmppi-toc.htm.

